# Efficacy of brexpiprazole on caregiver‐identified bothersome agitation symptoms that influence the decision to transfer to long‐term care: a 24‐week *post hoc* analysis of patients with dementia due to Alzheimer’s disease

**DOI:** 10.1002/alz70861_108617

**Published:** 2025-12-23

**Authors:** Anton P Porsteinsson, Sanjeda R Chumki, Anton M Palma, David Wang, Pedro Such, C Brendan Montano

**Affiliations:** ^1^ Alzheimer’s Disease Care, Research and Education Program (AD‐CARE), Rochester, NY USA; ^2^ Otsuka Pharmaceutical Development & Commercialization Inc., Princeton, NJ USA; ^3^ Lundbeck LLC, Deerfield, IL USA; ^4^ H. Lundbeck A/S, Valby, Copenhagen Denmark; ^5^ Connecticut Clinical Research, Cromwell, CT USA

## Abstract

**Background:**

Patients with Alzheimer’s dementia can experience a wide range of agitation symptoms. In a caregiver survey that assessed the Cohen‐Mansfield Agitation Inventory (CMAI), agitation symptoms considered “most bothersome”, and which influenced caregiver decisions to transfer to long‐term care, included aggressive behaviors (e.g., cursing or verbal aggression, hitting), verbally agitated behaviors (e.g., constant unwarranted requests for attention/help, repetitive sentences/questions), and physically non‐aggressive behaviors (e.g., pacing/aimless wandering). Brexpiprazole (an atypical antipsychotic with noradrenergic, serotonergic, and dopaminergic activity) is the only FDA‐approved treatment for agitation associated with dementia due to Alzheimer’s disease. This *post hoc* analysis explored effects of brexpiprazole on individual agitation symptoms over 24 weeks, focusing on the most bothersome symptoms.

**Method:**

Data were included for patients who received brexpiprazole 2 or 3 mg/day in a Phase 3, 12‐week, randomized, placebo‐controlled trial in patients with agitation associated with dementia due to Alzheimer’s disease (ClinicalTrials.gov: NCT03548584 [Trial 213]) and a 12‐week single‐arm extension trial (NCT03594123 [Trial 182]). Agitation symptom frequency was evaluated using the CMAI, which comprises 29 symptoms (items) each scored from 1 (never occurs) to 7 (occurs a few times an hour). In subgroups of patients who experienced a particular symptom at least weekly at baseline (CMAI item score ≥3), mean change in that item score from baseline to Week 12, and from Week 12 to Week 24, was calculated.

**Result:**

Overall, 159 patients were included across subgroups. From baseline to Week 12, all symptoms in the aggressive, verbally agitated, and physically non‐aggressive domains, including those most bothersome to caregivers and/or associated with transfer to long‐term care, reduced in frequency. From Week 12 to Week 24, symptoms considered most bothersome and/or associated with transfer to long‐term care showed further numerical improvement; other symptoms also improved, or stabilized.

**Conclusion:**

Treatment with brexpiprazole 2 or 3 mg/day over 24 weeks was associated with continued and sustained benefits on agitation symptoms that are considered most bothersome to caregivers, and that influence caregiver decisions to transfer patients to long‐term care.

